# Pathological Correlation of a Cardiac Mass with Multimodality Imaging

**DOI:** 10.1155/2023/7352934

**Published:** 2023-04-18

**Authors:** Sumit Sohal, Farida A. Tanko, Esad Vucic, Sergio Waxman, Suresh Gupta, Billie Fyfe-Kirschner

**Affiliations:** ^1^Division of Cardiology, Department of Internal Medicine, RWJBH Newark Beth Israel Medical Center, 201 Lyons Ave, Newark, NJ 07112, USA; ^2^Department of Pathology, Robert Wood Johnson University Hospital, 125 Paterson St, New Brunswick, NJ 08901, USA; ^3^Department of Pathology, RWJBH Newark Beth Israel Medical Center, 201 Lyons Ave, Newark, NJ 07112, USA

## Abstract

Cardiac masses are rarely encountered in clinical practice and can lead to severe hemodynamic consequences. In addition to clinical cues, noninvasive modalities can play an important role in characterization of these masses and therefore their diagnosis and management planning. Here in this case report, we describe the use of various forms of noninvasive imaging techniques to narrow the differential diagnosis and form an operative plan for a cardiac mass later identified as a benign myxoma originating from the right ventricle on histological examination.

## 1. Introduction

Cardiac masses were traditionally identified only during autopsy findings; however, with the advent of widely available noninvasive imaging techniques, their diagnosis has become easier and their management a reality [1]. Advancements in these imaging modalities have led to tissue characterization and differentiation between different types of pathologies prior to their histological examination. In this case report, we describe the use of multimodality imaging techniques to differentiate between various forms of cardiac masses and how these helped in the diagnosis of the mass from the right ventricle.

## 2. Case Presentation

**Figure 1:**
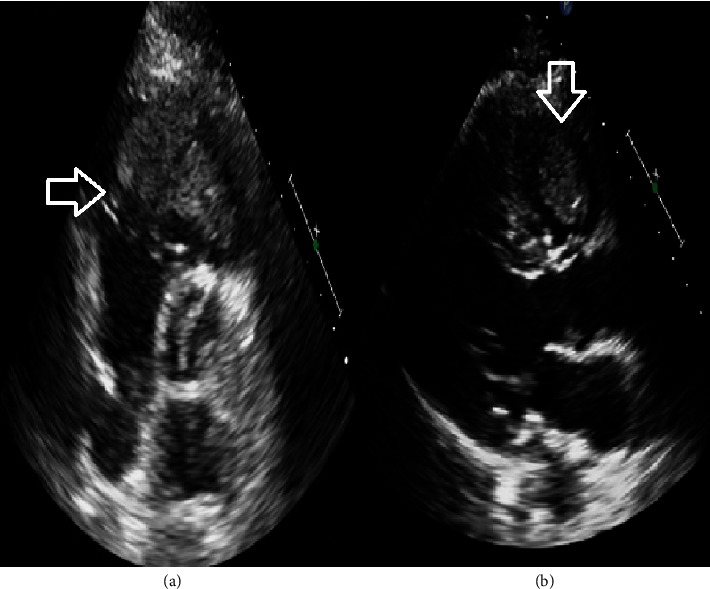
Transthoracic echocardiogram showing RV mass. (a) Parasternal short axis view demonstrates the mass (white arrow) extending from the undersurface of the tricuspid annulus and traversing the pulmonic valve. (b) Redemonstration of RV mass (white arrow) in the parasternal long axis view. Abbreviations: RV: right ventricle.

**Figure 2:**
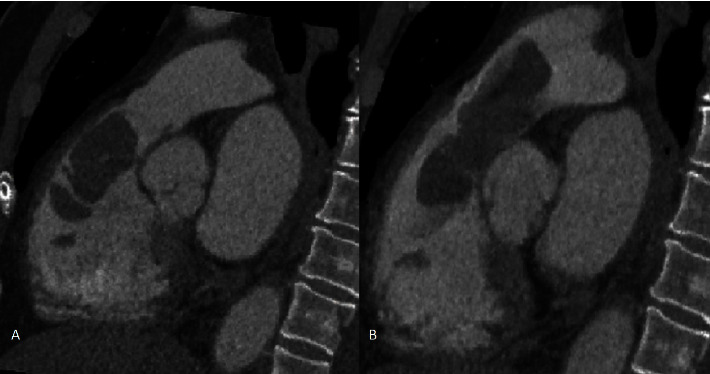
Gated computed tomographic angiography of the chest showing the mass in the RV (a) traversing the pulmonic valve into the main pulmonary artery. (b) Abbreviations: RV: right ventricle.

**Figure 3:**
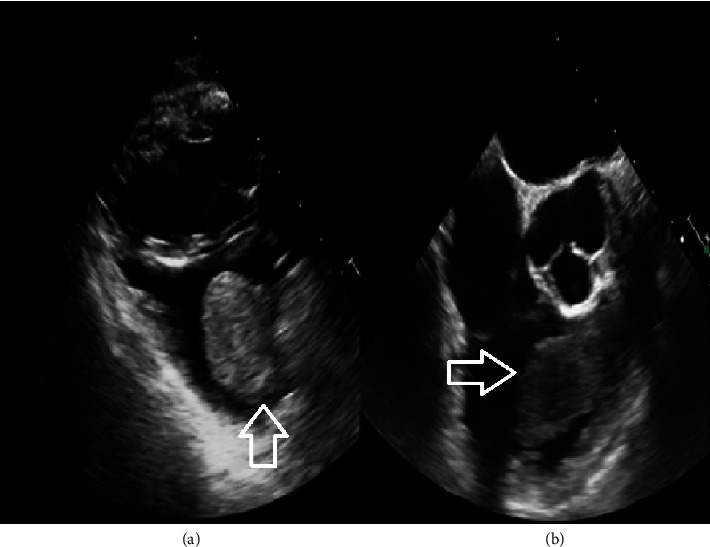
Transesophageal echocardiographic views of the RV mass. (a) Shows trans-gastric views which demonstrates the attachment of the mass to the undersurface of tricuspid annulus adjacent to the anterior tricuspid leaflet (white arrow). (b) Shows the short axis view where the RV mass (white arrow) is visualized traversing the pulmonary valve.

**Figure 4:**
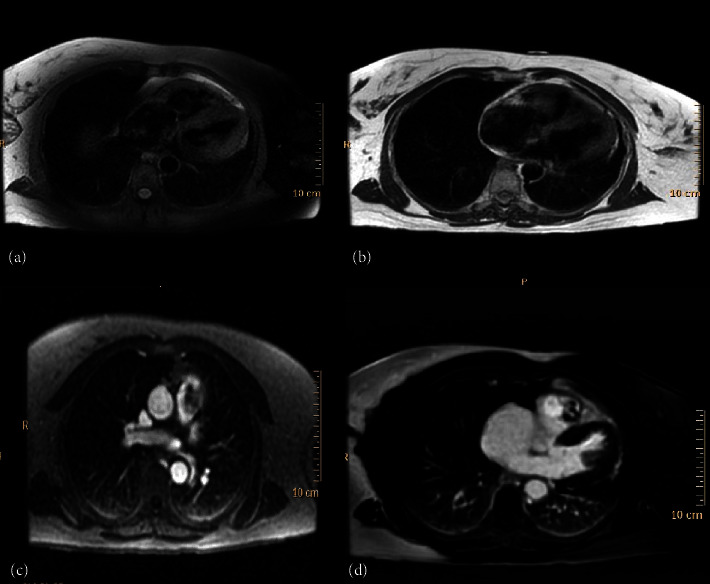
Cardiac magnetic resonance imaging shows RV mass. (a) shows RV mass, which is hypointense on T1 sequence, isointense on T2 (b), (c) shows poor first pass perfusion, and (d) shows heterogeneous uptake on late gadolinium enhancement. Abbreviations: RV: right ventricle.

**Figure 5:**
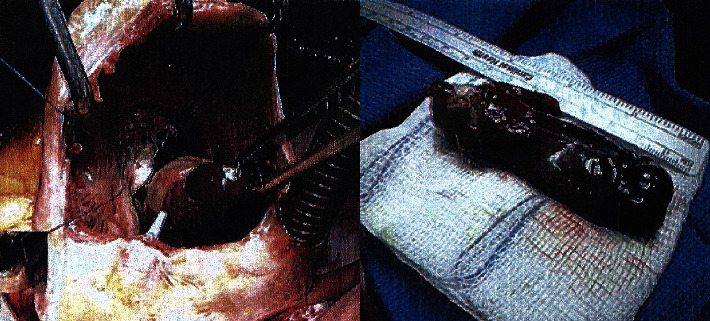
Intraoperative view shows a soft and friable red gelatinous mass being retrieved from the right atrium.

A female patient in her 60s with a medical history of hypertension followed up with her primary care physician for an annual physical examination. Upon evaluation, the patient denied any complaints, and a review of the system was negative for any symptoms. Cardiac auscultation revealed a regular rate and rhythm with a III/VI systolic murmur best heard at the apex. The rest of her examination and vitals were normal. The electrocardiogram demonstrated normal sinus rhythm with no abnormalities. A transthoracic echocardiogram (TTE) was ordered to evaluate the etiology of the systolic murmur which demonstrated moderate concentric left ventricular hypertrophy with normal cavity size and ejection fraction. A mobile, pedunculated mass was seen extending from the undersurface of the tricuspid annulus and traversing the pulmonic valve into the main pulmonary artery with mild right ventricular (RV) dilatation and hypokinesia (Figure 1). A gated computed tomographic angiography (CTA) of the chest confirmed the RV mass with no evidence of pulmonary embolism or extra-cardiac extension (Figure 2 and Video SF1). Patient was admitted for further evaluation and underwent a transesophageal echocardiogram (TEE), which redemonstrated a large mobile and pedunculated mass measuring 8 by 3 cm in the RV protruding into the pulmonary artery (PA) during systole. The trans-gastric views demonstrated the attachment of the mass to the undersurface of the tricuspid annulus adjacent to the anterior tricuspid leaflet via a narrow stalk measuring 1.4 cm and extending 3-4 cm into the main pulmonary artery (Figure 3). The mass appeared lobulated with partial enhancement on injection of commercial echo contrast, thus helping us to exclude a thrombus. On cardiac magnetic resonance imaging (CMR), the mass was hypointense on the T1 sequence, had poor uptake on first-pass perfusion, and a heterogeneous uptake was on late gadolinium enhancement, which reflective of a benign tumor like myxoma (Figure 4 and Video SF2). Given the large size, likely benign nature, and location of the tumor, the patient underwent surgical resection of the mass. Intraoperatively, the tumor was found to be soft and friable, extending into the PA, requiring dissection of the main PA to ensure complete retrieval of the mass into the right atrium (Figure 5). A partial tricuspid valve (TV) repair with an annuloplasty was also done. She had an uneventful course postoperatively. Patient has remained asymptomatic with no recurrence on follow-up echocardiogram 18 months after surgery.

## 3. Pathological Findings

**Figure 6:**
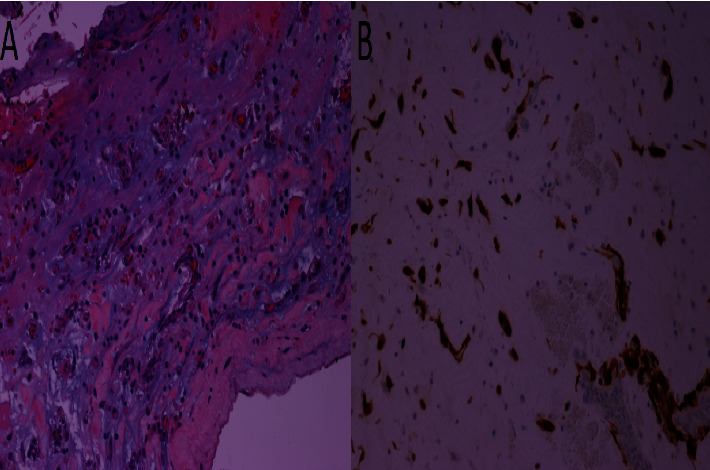
(a) Hematoxylin and eosin stain at 20x magnification with basophilic matrix, few spindle cells with no mitotic activity. (b) Calretinin immunoreactivity at 20x.

Pathology confirmed a smooth glistening gelatinous tan-red mass measuring 7.0 × 2.0 × 3.0 cm. Microscopically, the lesion consisted of bland cells in a basophilic matrix with a few spindle cells. No lipoblast or mitotic activity was noted. Ancillary testing using immunohistochemical stains was positive for calretinin, supporting the diagnosis of myxoma with excised margins negative for tumor (Figure 6).

## 4. Discussion

Cardiac masses are rare and may include tumors, thrombi, vegetations, or calcific lesions which unlike other organs can have severe hemodynamic consequences due to obstruction, embolism, electrical, or mechanical dysfunction [2]. Cardiac myxomas are the most common benign primary cardiac neoplasm seen in adults. The majority arise from the left atrium attached to the interatrial septum with less than 5% ventricular in origin [3]. In addition to clinical cues, noninvasive cardiac imaging plays a major role in identification and diagnosis of cardiac masses. Basic measures such as TTE and TEE can help in the assessment of size, location, and mobility of a mass, and the added feature of 3-dimensional echocardiography can provide more accurate anatomical relationships. The addition of contrast agents can help assess the perfusion of the mass and differentiate between a thrombus (avascular) and a tumor (vascular) [4]. In our patient the partially contrast-enhancing nature of the heterogeneous mass helped differentiate it as a tumor from a thrombus. TEE also helped in delineating anatomical relationships and guiding surgical access planning for retrieval of the mass.

Cardiac MRI with its tissue characterization and ability to provide information about extracardiac structures can help in assessing cardiac masses. First-pass perfusion to assess vascularity (high for vascular structures) and late gadolinium enhancement (absent in thrombus) can help to differentiate tumors from thrombus. T1 and T2 mapping along with other features can also help to differentiate between types of tumors [4, 5]. Cardiac CT can be used for more precise assessment of anatomical relations including extra-cardiac involvement but may not be required if other noninvasive measures are available. In our patient, heterogeneous late gadolinium enhancement, low first pass perfusion, and partial enhancement on injection of commercial echo contrast helped to exclude thrombus and identify the mass as a likely benign tumor as compared to a highly vascular lesion which has high first pass perfusion.

This case therefore not only describes a rare case of an incidental myxoma from the right ventricle but also shows how multimodality noninvasive imaging can be used to differentiate masses, aid in the management of these patients, and provide excellent correlation with histological diagnosis.

## 5. Conclusion

The imaging techniques guide in preoperative diagnosis and management of patients with an unidentified cardiac mass. Tissue characterization through multimodality imaging may provide an excellent correlation to the pathological diagnosis of a mass.

## Data Availability

The data used to support the findings of this study are included within the article.

## Supplementary Materials

Supplementary Materials SF1: Cine images showing the mass in the RV traversing the pulmonic valve into the main pulmonary artery on computed tomographic image of the chest. SF2: Cine images of magnetic resonance imaging showing the RV mass traversing the pulmonic valve.
